# Dissecting the genome of star fruit (*Averrhoa carambola* L.)

**DOI:** 10.1038/s41438-020-0306-4

**Published:** 2020-06-01

**Authors:** Yannan Fan, Sunil Kumar Sahu, Ting Yang, Weixue Mu, Jinpu Wei, Le Cheng, Jinlong Yang, Ranchang Mu, Jie Liu, Jianming Zhao, Yuxian Zhao, Xun Xu, Xin Liu, Huan Liu

**Affiliations:** 1 State Key Laboratory of Agricultural Genomics, China National GeneBank, BGI-Shenzhen, 518120 Shenzhen, China; 2 BGI-Yunnan, BGI-Shenzhen, 650106 Kunming, China; 3 Forestry Bureau of Ruili, Yunnan Dehong, 678600 Ruili, China; 4 Chinese Academy of Forestry, Beijing, China; 5 Guangdong Provincial Key Laboratory of Genome Read and Write, 518120 Shenzhen, China; 6 BGI-Fuyang, BGI-Shenzhen, 236009 Fuyang, China; 7 Department of Biology, University of Copenhagen, Copenhagen, Denmark

**Keywords:** Plant evolution, Plant genetics

## Abstract

*Averrhoa carambola* is commonly known as star fruit because of its peculiar shape, and its fruit is a rich source of minerals and vitamins. It is also used in traditional medicines in countries such as India, China, the Philippines, and Brazil for treating various ailments, including fever, diarrhea, vomiting, and skin disease. Here, we present the first draft genome of the Oxalidaceae family, with an assembled genome size of 470.51 Mb. In total, 24,726 protein-coding genes were identified, and 16,490 genes were annotated using various well-known databases. The phylogenomic analysis confirmed the evolutionary position of the Oxalidaceae family. Based on the gene functional annotations, we also identified enzymes that may be involved in important nutritional pathways in the star fruit genome. Overall, the data from this first sequenced genome in the Oxalidaceae family provide an essential resource for nutritional, medicinal, and cultivational studies of the economically important star-fruit plant.

## Introduction

The star-fruit plant (*Averrhoa carambola* L.), a member of the Oxalidaceae family, is a medium-sized tree that is distinguished by its unique, attractive star-shaped fruit (Supplementary Fig. 1). *A. carambola* is widely distributed around the world, especially in tropical countries such as India, Malaysia, Indonesia, and the Philippines, and is considered an important species; thus, it is extensively cultivated in Southeast Asia and Malaysia^1,2^. It is also a popular fruit in the markets of the United States, Australia, and the South Pacific Islands^3^. Star fruits have a unique taste, with a slightly tart, acidic (in smaller fruits) or sweet, mild flavor (in large fruits). Star fruit is a good source of various minerals and vitamins and is rich in natural antioxidants such as vitamin C and gallic acid. Moreover, the presence of high amounts of fiber in these fruits aids in absorbing glucose, retarding glucose diffusion into the bloodstream and controlling the blood glucose concentration.

In addition to its use as a food source, star fruit is utilized as an herb in India, Brazil, and Malaysia and is widely used in traditional Chinese medicine preparations^4^ as a remedy for fever, asthma, headache, and skin diseases^5^. Several studies have demonstrated the presence of various phytochemicals, such as saponins, flavonoids, alkaloids, and tannins, in the leaves, fruits, and roots of star-fruit plants^6,7^; these compounds are known to confer antioxidant and specific healing properties. A study by Cabrini et al.^5^ indicated that the ethanolic extract from *A. carambola* is highly effective in minimizing the symptoms of ear swelling (edema) and cellular migration in mice. A flavonoid compound (apigenin-6-C-β-fucopyranoside) isolated from *A. carambola* leaves showed anti-hyperglycemic action in rats, and might show potential for use in the treatment and prevention of diabetes^8^. Moreover, 2-dodecyl-6-methoxycyclohexa-2,5-diene-1,4-dione (DMDD) extracted from the roots of *A. carambola* exhibits potential benefits in the treatment of obesity, insulin resistance, and memory deficits in Alzheimer’s disease^9,10^.

Although *A. carambola* plays very significant roles in traditional medicine applications, there are very limited studies on *A. carambola* at the genetic level, mainly due to a lack of genome information. Therefore, filling this genomic gap will help researchers to fully explore and understand this agriculturally important plant. As a part of the 10KP project^11,12^, the draft genome of *A. carambola* collected from the Ruili Botanical Garden in Yunnan, China, was assembled in this study using an advanced 10X genomics technique to further elucidate the evolution of the Oxalidaceae family. A fully annotated genome of *A. carambola* will serve as a foundation for pharmaceutical applications of the species and the improvement of breeding strategies for the star-fruit plant.

## Results

### Genome assembly and evaluation

Based on k-mer analysis, a total of 35,655,285,391 k-mers were used, with peak coverage of 75. The *A. carambola* genome was estimated to be ~475 Mb in size (Supplementary Fig. 2). To perform genome assembly, a total of 156 Gb of clean reads were utilized by Supernova v2.1.1^13^. The final assembly contained 69,402 scaffold sequences, with an N50 of 2.76 Mb, and 78,313 contig sequences, with an N50 of 44.84 Kb, for a total assembly size of 470.51 Mb (Table 1). Completeness assessment was performed using Benchmarking Universal Single-Copy Orthologs (BUSCO) version 3.0.1^14^ with Embryophyta odb9. The results showed that 1327 (92.20%) of the expected 1440 conserved plant orthologs were detected as complete (Supplementary Table 1). To further evaluate the completeness of the assembled genome, we performed short read mapping using clean raw data with BWA-MEM software^15^. In total, 943,278,896 (99.12%) reads could be mapped to the genome, and 88.13% of them were properly paired (Supplementary Table 2).

**Table 1:** Statistics of genome assembly.

	Contig		Scaffold	
	Size (bp)	Number	Size (bp)	Number
N90^a^	5420	12,457	7033	3988
N80	13,875	7548	39,210	608
N70	23,325	5165	34,6109	131
N60	33,460	3619	1,307,770	60
N50	44,841	2503	2,757,598	35
Longest	717,770	–	14,768,062	–
Total size	431,262,337	–	470,508,511	–
Total number (≥2 kb)	–	18,820	–	10,777
Total number (≥100 bp)	–	78,313	–	69,402

^a^N*xx* length is the maximum length (*L*) such that *xx*% of all nucleotides lie within contigs (or scaffolds) with a size of at least *L*.

### Genome annotation

A total of 68.15% of the assembled *A. carambola* genome was composed of repetitive elements (Supplementary Table 3). Among these repetitive sequences, LTRs were the most abundant, accounting for 61.64% of the genome. DNA class repeat elements represented 4.19% of the genome; LINE and SINE classes accounted for 0.28% and 0.016% of the assembled genome, respectively. For gene prediction, we combined homology-based and de novo-based approaches and obtained a non-redundant set of 24,726 gene models with 4.11 exons per gene on average. The gene length was 3457 bp on average, while the average exon and intron lengths were 215 and 827 bp, respectively. The gene model statistical data compared with seven other closely related species are shown in Supplementary Fig. 3. To evaluate the completeness of the gene models for *A. carambola*, we used BUSCO with Embryophyta odb9. A total of 1281 (88.9%) complete orthologs were detected from the predicted star fruit gene sets (Supplementary Table 4).

Functions were assigned to 16,490 (66.69%) genes. These protein-coding genes were then subjected to further exploration against the KEGG, NR, and COG protein sequence databases^16^, in addition to the SwissProt and TrEMBL databases^17^, and InterProScan^18^ was finally used to identify domains and motifs (Supplementary Table 5, Supplementary Fig. 4). Noncoding RNA genes in the assembled genome were also annotated. We predicted 759 tRNA, 1341 rRNA, 90 microRNA (miRNA), and 2039 small nuclear RNA (snRNA) genes in the assembled genome (Supplementary Table 6).

Since star fruit is an important cultivated plant, the identification of disease resistance genes was one of the focuses of our study. Nucleotide-binding site (NBS) genes play an important role in pathogen defense and the cell cycle. We identified a total of 80 non-redundant NBS-encoding orthologous genes in the star fruit genome (Supplementary Table 7). Among these genes, TIR (encoding the toll interleukin receptor) motif was found to be significantly smaller than in other eudicot plants, except for cocoa. Unlike other plants, the leucine-rich repeat (LRR) motif was not the most or second most common motif in the NBS gene family in star fruit^19^.

### Genome evolution

**Fig. 1:**
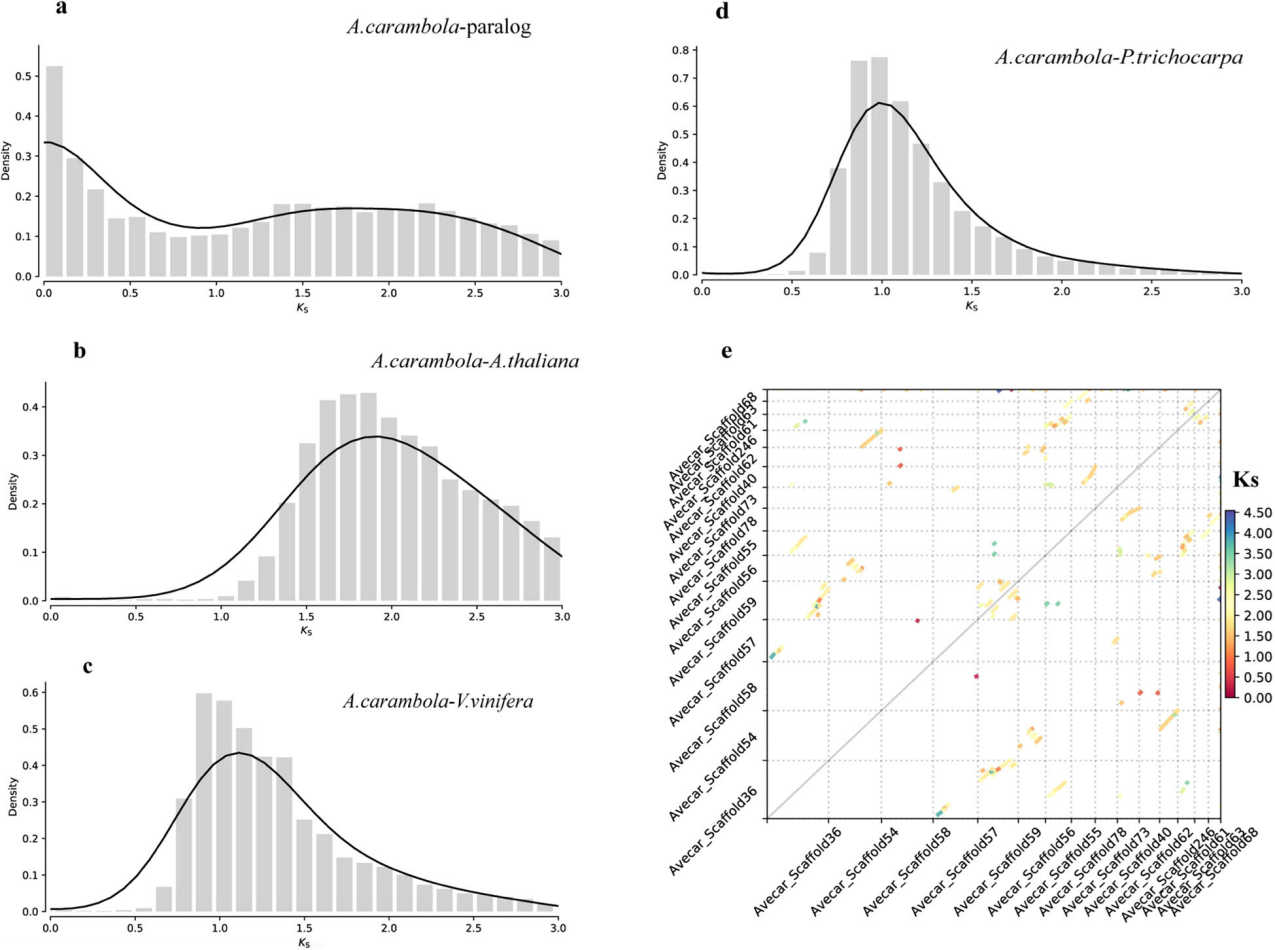
The analysis of *A. carambola* whole*-*genome duplications. The distribution of synonymous substitution rate (Ks) distance values observed for **a** *A. carambola-*paralog, **b** *A. carambola–A. thaliana* ortholog, **c** *A. carambola–V. vinifera* ortholog, and **d** *A. carambola–P. trichocarpa* ortholog. **e** The *A. carambola* paralog colinear blocks are colored according to Ks values.

The characterization of the star fruit genome can provide necessary data for further analyzing the evolutionary history of Oxalidaceae. A γ whole-genome triplication event affected over 75% of extant angiosperms and was associated with the early diversification of the core eudicots. To investigate evolutionary events at the genomic level in star fruit, we identified 1134 paralogous gene families on the basis of the 24,726 gene models. The synonymous substitution rates (Ks) in the duplicated genes (Ks = 1.9) suggested that an ancient γ event occurred in star fruit (Fig. 1a). Furthermore, we assessed the intergenomic collinearity among the *Arabidopsis*^20^, poplar^21^, and grape^22^ genomes and identified relationships among star fruit orthologues. The mean Ks values from the one-to-one orthology analysis of star fruit in relation to *Arabidopsis*, poplar, and grape were 1.8, 1.0, and 1.2, respectively (Fig. 1b–d). The results confirmed the shared ancient whole-genome duplications (WGDs) event between the four species. Moreover, we generated whole-genome syntenic dotplots of star fruit based on the Ks value (Fig. 1e). Over 50% of the syntenic blocks shared a Ks rate between 1.0 and 2.0, and only ~10% of the gene pairs exhibited a Ks below 1.0, which indicated that no recent WGDs have occurred in the star fruit genome.

### Gene family analysis and phylogenetic tree

**Fig. 2:**
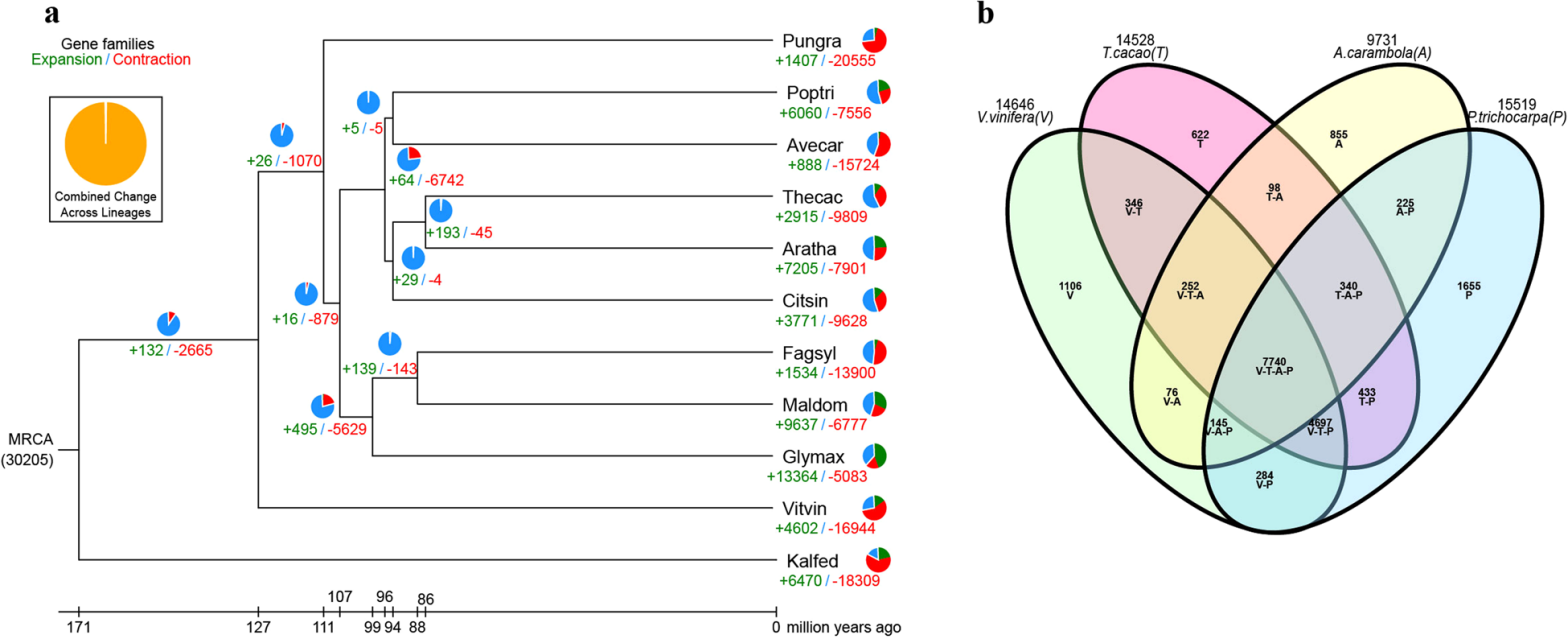
Gene family analysis and phylogenetic tree construction. **a** Phylogenetic tree showing the sizes of expanded and contracted gene families. Pungra: *P. granatum;* Poptri: *P. trichocarpa;* Avecar: *A. carambola;* Thecac: *T. cacao;* Aratha: *A. thaliana;* Citsin: *C. sinensis;* Fagsyl: *F. sylvatica;* Maldom: *M. domestica;* Glymax: *G. max*; Vitvin: *V. vinifera*; Kalfed: *K. fedtschenkoi*. **b** Venn diagram of the number of shared gene families within *A. carambola*, *V. vinifera*, *T. cacao*, and *P. trichocarpa*.

We performed *A. carambola* gene family analysis using OrthoMCL software^23^ with protein and nucleotide sequences from *A. carambola* and 10 other plant species (*A. thaliana*, *C. sinensis*, *F. sylvatica*, *G. max*, *K. fedtschenkoi*, *M. domestica*, *P. granatum*, *P. trichocarpa*, *T. cacao*, *V. vinifera*) based on an all-versus-all BLASTP alignment with an *E*-value cutoff of 1e−05. The 24,726 predicted protein-coding genes in *A. carambola* were assigned to 9731 gene families consisting of 15,301 genes, while 9425 genes were not organized into groups and were unique to *A. carambola* (Supplementary Table 8, Fig. 2b). In total, 163 single-copy orthologs corresponding to the 11 species were extracted from the clusters and used to construct the phylogenetic tree. The constructed tree topology supported the APG IV^24^ system in which Oxalidales (*A. carambola*) and Malpighiales (*P. trichocarpa*) are sister clades that belong to the same cluster (rosids). Based on the phylogenetic tree, *A. carambola* was estimated to have separated from *P. trichocarpa, V. vinifera*, and *K. fedtschenkoi* approximately 94.5, 110.2, and 126.3 Mya, respectively (Supplementary Fig. 5).

We also analyzed the expansion and contraction of the gene families between species using CAFÉ^25^. The results showed that 888 gene families were substantially expanded and that 15,724 gene families were contracted in *A. carambola* (Fig. 2a). In total, 2916 and 6057 genes identified in *A. carambola* came from expanded and contracted families, respectively, where contraction was approximately two times more common than expansion.

Gene ontology (GO) and KEGG functional enrichment analyses were subsequently performed for all expanded gene families. The KEGG pathway enrichment analysis results are shown in Table 2, and the GO-enrichment results are listed in Supplementary Table 9. In a previous study, several flavonoids were isolated from the fresh fruit of *A. carambola*, which are known to reduce harmful inflammation^26^. In our study, the flavonoid biosynthesis pathway was found to be significantly enriched among the expanded families. Terpenoids are yet another important type of compound that has been isolated from star fruit^27^ and has been proven to exhibit anti-inflammatory activities. *A. carambola* likely synthesizes terpenoids via the diterpenoid biosynthesis pathway.

**Table 2:** Enriched KEGG pathways of unique genes of *A. carambola* showing expansion.

Pathway ID	KEGG description	Adjusted *P*-value (≤0.05)	Number of genes
map03020	RNA polymerase	3.51E–09	30
map00904	Diterpenoid biosynthesis	1.00E–05	18
map00240	Pyrimidine metabolism	3.16E–05	34
map01100	Metabolic pathways	7.17E–05	234
map00901	Indole alkaloid biosynthesis	7.17E–05	15
map00230	Purine metabolism	7.17E–05	36
map00565	Ether lipid metabolism	0.00014092	14
map01110	Biosynthesis of secondary metabolites	0.00038039	142
map02010	ABC transporters	0.00080624	20
map00902	Monoterpenoid biosynthesis	0.00116462	9
map00460	Cyanoamino acid metabolism	0.00270665	18
map00941	Flavonoid biosynthesis	0.03063434	17
map00190	Oxidative phosphorylation	0.0354555	19
map00940	Phenylpropanoid biosynthesis	0.0354555	32
map00062	Fatty acid elongation in mitochondria	0.0354555	7
map00563	Glycosylphosphatidylinositol(GPI)-anchor biosynthesis	0.03937265	8
map00040	Pentose and glucuronate interconversions	0.0426558	21

### Genes specifically involved in star fruit nutrition pathways

Star fruit is an excellent source of various minerals and vitamins, especially natural antioxidants, such as l-ascorbic acid (vitamin C) and riboflavin (vitamin B_2_)^1,26^. Through the ortholog searches in KEGG pathways, we identified enzymes that are potentially involved in the vitamin C and vitamin B_2_ biosynthesis pathways in *A. carambola*.

**Fig. 3:**
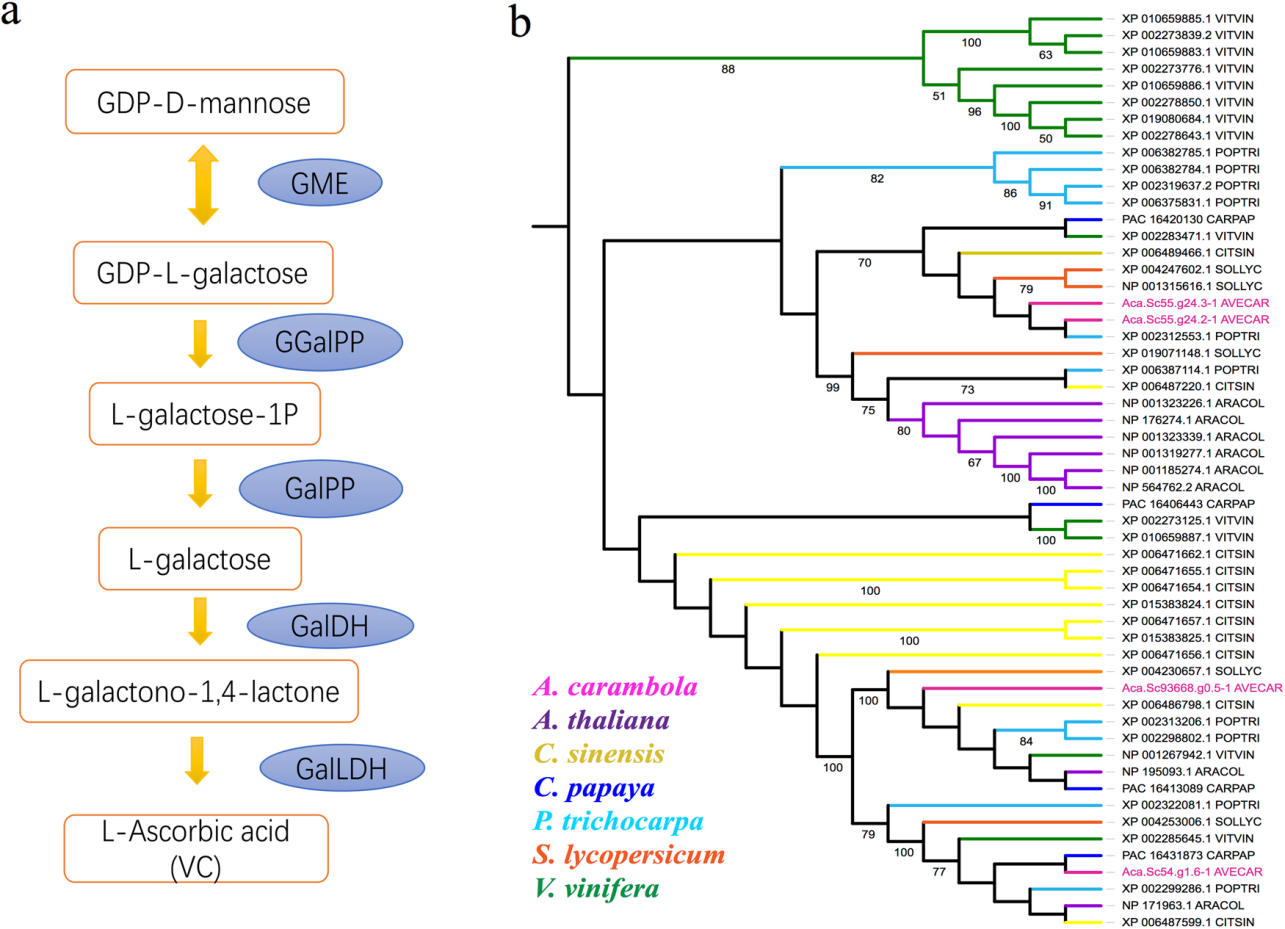
Genes involved in vitamin C metabolism. **a** A proposed model for l-ascorbic acid biosynthesis pathways in star fruit. Genes identified as being involved in the pathways are shown in blue circles. **b** Phylogenetic analysis of the GalDH gene family in *A. carambola* (rose red), *A. thaliana* (purple), *C. sinensis* (yellow), *C. papaya* (blue), *P. trichocarpa* (sky blue), *S. lycopersicum* (orange), and *V. vinifera* (green).

In a previous report, a major component of plant ascorbate was reported to be synthesized through the l-galactose pathway^28^, in which GDP-d-mannose is converted to l-ascorbate through four successive intermediates, as summarized in Fig. 3a. Laing et al. ^29^ reported the identification of l-galactose guanylyltransferase-encoding homologous genes from *Arabidopsis* and kiwifruit that encode enzymes that catalyze the conversion of GDP-l-galactose to l-galactose-1-P. In this study, five necessary enzymes (GalDH, GalLDH, GGalPP, GalPP, and GME) involved in the vitamin C pathway were identified, suggesting the possibility of ascorbic acid synthesis in star fruit (Table 3). For l-galactose dehydrogenase (GalDH), we identified four paralogous genes in the star fruit genome. The copy number of GalDH genes in star fruit is close to that in tomato (5) and papaya (4) but approximately half that in other species (10 in poplar, 11 in orange, 8 in Arabidopsis, and 13 in grape, Supplementary Table 12). Further evolutionary analysis showed three clusters in the phylogenetic tree, and the most ancient cluster comprised all the grape genes. Among the other two sister clusters, one is ancient and comes from poplar, including four genes, and the other is closer to orange, including seven genes. The four genes in star fruit are divided into two clusters and were recently separated from their ancestors (Fig. 3b).

**Table 3:** List of genes involved in the vitamin C and vitamin B_2_ pathways.

Pathway	Enzyme	Description	Copy numbers	Gene ID	Protein (AA)
Vitamin C pathway	GalDH	l-galactose dehydrogenase	4	Aca.sc093668.g0.5	332
				Aca.sc000054.g1.6	334
				Aca.sc000055.g24.2	351
				Aca.sc000055.g24.3	594
	GalLDH	l-galactono-1,4-lactone dehydrogenase	5	Aca.sc000059.g19.35	601
				Aca.sc000229.g0.29	603
				Aca.sc000078.g43.5	598
				Aca.sc100684.g0.3	99
				Aca.sc102574.g0.3	99
	GGalPP	GDP-l-galactose phosphorylase	3	Aca.sc150475.g0.5	275
				Aca.sc150564.g0.4	288
				Aca.sc098705.g0.4	410
	GalPP	l-galactose-1-phosphate phosphatase	2	Aca.sc006151.g1	181
				Aca.sc000246.g13.57	360
	GME	GDP-d-mannose-3′,5′-epimerase	2	Aca.sc096116.g0.5	383
				Aca.sc000061.g13.42	383
Vitamin B_2_pathway	RIB3	3,4-dihydroxy 2-butanone 4-phosphate synthase	3	Aca.sc000063.g38.15	545
				Aca.sc000056.g65.27	572
				Aca.sc000071.g11.34	590
	RIB4	6,7-dimethyl-8-ribityllumazine synthase	1	Aca.sc023103.g76.2	185
	RIB5	riboflavin synthase	1	Aca.sc000036.g2.16	343
	RFK	riboflavin 5′-phosphotransferase	1	Aca.sc000058.g77.42	539
	FLAD1	FMN adenylyltransferase	1	Aca.sc000058.g25.58	520

*GalDH* l-galactose dehydrogenase, *GalLDH* l-galactono-1,4-lactone dehydrogenase, *GGalPP* GDP-l-galactose phosphorylase, *GalPP* l-galactose-1-phosphate phosphatase, *GME* GDP-d-mannose-3′,5′-epimerase.

**Fig. 4:**
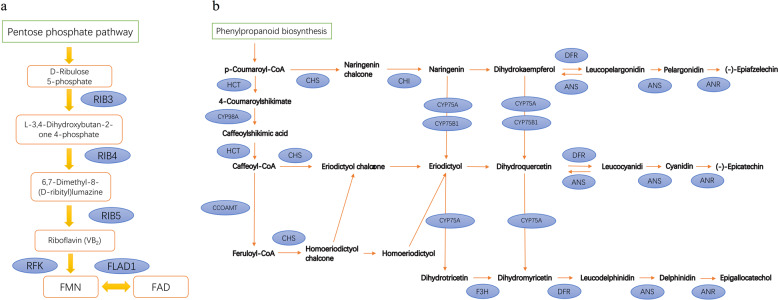
Identification of genes involved in the (**a**) riboflavin and (**b**) flavonoid biosynthesis pathways. Genes identified as being involved in the two pathways are shown in blue circles. FLAD1 FMN adenylyltransferase, RIB3 3,4-dihydroxy 2-butanone 4-phosphate synthase, RIB4 6,7-dimethyl-8-ribityllumazine synthase, RIB5 riboflavin synthase, RFK riboflavin 5′-phosphotransferase, ANR anthocyanidin reductase, ANS leucoanthocyanidin dioxygenase, CCOAMT caffeoyl-CoA O-methyltransferase, CHI chalcone isomerase, CHS chalcone synthase, CYP75A flavonoid 3′,5′-hydroxylase, CYP75B1 flavonoid 3′-monooxygenase, CYP98A coumaroylquinate 3′-monooxygenase, DFR flavanone 4-reductase, F3H naringenin 3-dioxygenase, HCT shikimate O-hydroxycinoyltransferase.

We also identified possible enzymes involved in the riboflavin (vitamin B_2_) biosynthesis pathway in star fruit (Fig. 4a, Table 3). Through catalysis by RIB3, RIB4, and RIB5, riboflavin can ultimately be produced from the d-ribulose 5-phosphate compound. Furthermore, in the investigation of the possible biosynthesis pathway of the special product oxalate in star fruit, we identified three enzymes, citrate synthase (CS), isocitrate lyase (aceA), and aconitate hydratase (ACO), that can potentially catalyze the transformation of oxalacetic acid to glyoxylate within the glyoxylate and dicarboxylate metabolism pathway (Supplementary Table 10).

In the *A. carambola* gene family analysis, the KEGG pathway enrichment analysis of the expanded gene families revealed that 17 genes participate in the flavonoid synthesis pathway (*P*-value = 0.03, Table 3). Previous studies have proved that flavonoids can be isolated from *A. carambola* and other plants from the Oxalidaceae family^1,9,26,30–33^. Here, we identified 11 enzymes that could be potentially involved in the flavonoid biosynthesis pathway (Fig. 4b, Supplementary Table 11). The two enzymes in the pathway showing the highest copy numbers are shikimate O-hydroxycinnamoyltransferase (HCT) and chalcone synthase (CHS), with 23 and 21 copies, respectively. Among the end-point products, apigenin, cyanidin, epicatechin, and quercetin have been extracted from the leaves, fruits or bark of *A. carambola* in previous studies^6,34–36^.

## Discussion

This study presents the first draft genome in the Oxalidaceae family. The sequenced species, *A. carambola* (star fruit), is widely cultivated and utilized as an edible fruit and serves as an important source of minerals and vitamins, in addition to presenting phytomedicinal properties. The assembled genome size was 470.51 Mb, with a scaffold N50 of 2.76 Mb. We cannot compare this genome size with those of other species in this family, but we found similar genome sizes in the closest order Malpighiales of 434.29 Mb in *Populus trichocarpa* and 350.62 Mb in *Ricinus communis*. However, the chromosome-level genome will be required to better understand the diploid character of this species.

In total, 24,726 gene models were identified from *A. carambola*. This gene number is relatively smaller than those from earlier reports for *A. thaliana, P. trichocarpa*, or *T. cacao*. The length distribution of the exons of the predicted star fruit proteins was consistent with those in other species, although the predicted intron and CDS lengths tended to be shorter than those in other species (Supplementary Fig. 3). The proportion of predicted genes containing an InterPro functional domain was 52.3%, and the proportion that could be aligned with the NCBI nr database (66.4%) was the highest among all databases. It is likely that *A. carambola* is the only species in the Oxalidaceae family whose genome has been assembled so far; there might be some evolutionarily unique genes in this family remaining to be annotated.

We subsequently performed gene family analysis together with the other 10 species and identified the significant expansion of 888 gene families containing 2916 unique genes in *A. carambola*. These genes were significantly enriched (*P*-value ≤ 0.05) within 28 GO classes, including 18 biological process, 2 cellular component, and 8 molecular function categories (Supplementary Table 6). The biological process of DNA binding contained the most genes (60) within the expanded families. Within the significantly enriched biological processes, the defense response to fungus might be related to the antimicrobial property of star fruits identified in previous studies^1^. On the other hand, we found that oxidoreductase activity was enriched in the molecular function GO class, which could be one of the potential reasons for the antioxidant activity of star fruits.

The genome evolution analysis indicated that star fruit only shared an ancient γ event, and no recent WGD was observed. In a genome evolution study of poplar, which belongs to Malpighiales, the existence of a hexaploidization event and recent duplication were reported^21^. This result could partially explain why star fruit has half the number of gene sets compared to poplar.

Moreover, among the enriched KEGG pathways, we identified enzymes involved in nutritional metabolic pathways, including the vitamin C, vitamin B_2_, oxalate, and flavonoid pathways. Although potential functional enzymes have been identified from the genome, these functional pathways should be verified by experimental studies in the future.

In summary, it can be expected that this draft genome will facilitate the elucidation of the development of specific important traits in star fruit plants, such as the biosynthesis pathways of pharmacologically active metabolites, and will contribute to the improvement of breeding strategies for star fruit plants.

## Materials and methods

### Plant materials and sequencing

Leaf samples of *A. carambola* were collected from the Ruili Botanical Garden, Yunnan, China. Genomic DNA was extracted by using the cetyl-triethylammonium bromide (CTAB) method^37^. 10X Genome sequencing was performed to obtain high-quality reads. High-molecular-weight (HMW) DNA was loaded onto a Chromium Controller chip with 10X Chromium reagents and gel beads, and the rest of the procedures were carried according to the manufacturer’s protocol^38^. Then, the BGISEQ-500 platform was used to produce 2 × 150 bp paired-end sequences, generating a total of 206.28 Gb of raw data. The raw reads were filtered using SOAPfilter v2.2 with the following parameters: “-q 33 -i 600 -p -l -f -z -g 1 -M 2 -Q 20”. After filtering low-quality reads, ~114.42 Gb of clean data (high-quality reads >Q35) remained for the next step.

### Estimation of *A. carambola* genome size

All 114.42 Gb clean reads obtained from the BGISEQ-500 platform were subjected to 17 kmer frequency distribution analysis with Jellyfish^39^ using the parameters “-k 17 -t 24”. The frequency graph was drawn, and the *A. carambola* genome size was calculated using the formula: genome size = k-mer_Number/Peak_Depth.

### De novo genome assembly

The linked read data were assembled using Supernova v2.1.1 software^13^ using the “--localcores = 24 --localmem=350 --max reads 280000000” parameter. To fill the scaffold gaps, GapCloser version 1.12^40^ was used with the parameters “-t 12 -l 155”. Finally, the total assembled length of the *A. carambola* genome was 470.51 Mb, with a scaffold N50 of 275.76 Kb and a contig N50 of 44.84 Kb.

### Repeat annotation

For transposable element annotation, RepeatMasker v3.3.0^41^ and RepeatProteinMasker v3.3.0^41^ were applied against Repbase v16.10^42^ to identify known repeats in the *A. carambola* genome. Tandem repeats were identified using Tandem Repeats Finder v4.07b^43^. De novo repeat identification was conducted using the RepeatModeler v1.0.5^44^ and LTR_FINDER v1.05^45^ programs, followed by RepeatMasker v3.3.0^41^ to obtain the final results.

### Gene prediction

Prior to the gene prediction analysis, we masked the repetitive regions of the genome. MAKER-P v2.31^46^ was utilized to predict protein-coding genes based on homology and de novo prediction evidence. For homology-based prediction, the protein sequences of *Theobroma cacao*, *Prunus persica*, *Prunus mume*, *Prunus avium*, *Populus trichocarpa*, *Populus euphratica*, and *Arabidopsis thaliana* were aligned against the *A. carambola* genome using BLAT^47^. Then, the gene structure was predicted using GeneWise^48^. To optimize different ab initio gene predictors, we constructed a series of training sets for de novo prediction data. Complete gene models according to homology evidence were picked for training with the Augustus tool^49^. Genemark-ES v4.21^50^ was self-trained using the default criteria. The first round of MAKER-P analysis was also run using the default parameters on the basis of the above evidence, with the exception of “protein2genome”, which was set to “1”, yielding only protein-supported gene models. SNAP^51^ was then trained with these gene models. The default parameters were used to run the second and final rounds of MAKER-P, generating the final gene models.

### Functional annotation

The predicted gene models were functionally annotated by aligning their protein sequences against the Kyoto Encyclopedia of Genes and Genomes (KEGG)^52^, Clusters of Orthologous Groups (COG)^16^, SwissProt^17^, TrEMBL, and National Center for Biotechnology Information (NCBI) non-redundant (NR) protein databases with BLASTP (*E*-value ≤ 1e–05). Protein motifs and domains were identified by comparing the sequences against various domain databases, including the PFAM, PRINTS, PANTHER, ProDom, PROSITE, and SMART databases, using InterProScan v5.21^18^. For ncRNA annotation, tRNA genes were identified with tRNAscan-SE v1.23^53^. For the identification of rRNA genes, we aligned the assembled data against the rRNA sequences of *A. thaliana* using BLASTN (*E*-value ≤ 1e–05). miRNAs and snRNAs were predicted by using INFERNAL^54^ software against the Rfam database^55^.

To classify the NBS domains in the star fruit protein sequences, we used hidden Markov models (HMM) to search for the Pfam NBS (NB-ARC) family PF00931 with an *E*-value cutoff of 1.0. To detect TIR domains, the amino acid sequences were also screened by using the HMM model Pfam TIR PF01582 (*E*-value ≤ 1.0). Moreover, to identify LRR motifs, we performed an HMM search for Pfam LRR models (*E*-value ≤ 1.0) against star fruit NBS-encoding amino acid sequences.

To compare the orthologous genes in the vitamin C, vitamin B_2,_ flavonoid and oxalate pathways between other plant species (*P. trichocarpa*, *C. sinensis*, *A. thaliana*, *S. lycopersicum*, *C. papaya*, and *V. vinifera*), we also annotated the protein sequences by aligning them against the KEGG database^52^ with BLASTP (*E*-value ≤ 1e–05) and then performed filtering according to Pfam domains annotated using InterProScan v5.21^18^.

### Genome evolution

The genome sequences used for orthology analysis were downloaded from NCBI (https://www.ncbi.nlm.nih.gov) for *A. thaliana* (GCA_000001735.2), *V. vinifera* (GCA_000003745.2), and *P. trichocarpa* (GCA_000002775.3). Next, we used wgd software^56^ to perform the Ks distribution analysis. The analyses of paralogous gene families and one-to-one orthologs were performed using the “wgd mcl” command. Then, the Ks distribution for a set of paralogous families or one-to-one orthologs was constructed using “wgd ksd”. Next, we applied the “wgd kde” command for the fitting of kernel density estimates (KDEs). Finally, the colinear blocks of the *A. carambola* paralog were identified by I-ADHoRe^57^ and colored according to their median Ks value.

### Gene family construction and phylogenetic analysis

For gene family analysis, OrthoMCL^23^ software was utilized to construct the orthologous gene families of all the protein-coding genes of *A. carambola* and other 10 sequenced plant species (*A. thaliana*, *C. sinensis*, *F. sylvatica*, *G. max*, *K. fedtschenkoi*, *M. domestica*, *P. granatum*, *P. trichocarpa*, *T. cacao*, *V. vinifera*). Before the application of OrthoMCL, BLASTP was used to find similar matches from different species with an *E*-value cutoff of 1e–05. The composition of the OrthoMCL clusters was used to calculate the total number of gene families.

Orthogroups that were present in a single copy in all species analyzed were selected and aligned using MAFFT v7.310^58^. Each gene tree was constructed by using RAxML v8.2.4^59^ with the GTRGAMMA model. To construct the species phylogenetic tree, a coalescent-based method in ASTRAL v4.10.4^60^ with 100 replicates of multilocus bootstrapping^61^ was used.

The divergence time between *A. carambola* and other species was estimated using MCMCTREE^59^ with the default parameters. The expansion and contraction of gene family numbers were predicted using CAFÉ^25^ by employing the phylogenetic tree and gene family statistics.

To further perform the phylogenetic analysis of the key enzyme GalDH in the vitamin C pathway, we annotated orthologous genes from six other plant species (*A. thaliana*, *C. papaya*, *C. sinensis*, *P. trichocarp*, *V. vinifera*, *S. lycopersicum*) using BLASTP with an *E*-value cutoff of 1e–05 to align coding sequences against the KEGG database. In total, 55 orthologous genes were used to generate a phylogenetic tree via the maximum likelihood (ML) method in RAxML v 8.2.4^59^, and 20 runs were included to identify an optimal tree using the GTRGAMMA substitution model and 100 nonparametric bootstrap replicates.

## Supplementary information

Supplementarial_materials

## Data Availability

The datasets generated and analyzed during the current study are available in the CNGB Nucleotide Sequence Archive (CNSA: https://db.cngb.org/cnsa). The raw sequencing data are under ID CNR0066625, and assembly data are under ID CNA0002506. All other data generated or analyzed during this study are included in this published article and its supplementary information files.
